# Response of the Diatom *Phaeodactylum tricornutum* to Photooxidative Stress Resulting from High Light Exposure

**DOI:** 10.1371/journal.pone.0038162

**Published:** 2012-06-01

**Authors:** Nuno Domingues, Ana Rita Matos, Jorge Marques da Silva, Paulo Cartaxana

**Affiliations:** 1 Centro de Biodiversidade, Genómica Integrativa e Funcional, Faculdade de Ciências da Universidade de Lisboa, Lisboa, Portugal; 2 Centro de Oceanografia, Faculdade de Ciências da Universidade de Lisboa, Lisboa, Portugal; University of Connecticut, United States of America

## Abstract

The response of microalgae to photooxidative stress resulting from high light exposure is a well-studied phenomenon. However, direct analyses of photosystem II (PSII) D1 protein (the main target of photoinhibition) in diatoms are scarce. In this study, the response of the diatom model species *Phaeodactylum tricornutum* to short-term exposure to high light was examined and the levels of D1 protein determined immunochemically. Low light (LL) acclimated cells (40 µmol photons m^−2^ s^−1^) subjected to high light (HL, 1,250 µmol photons m^−2^ s^−1^) showed rapid induction of non-photochemical quenching (NPQ) and *ca.* 20-fold increase in diatoxanthin (DT) concentration. This resulted from the conversion of diadinoxanthin (DD) to DT through the activation of the DD-cycle. D1 protein levels under LL decreased about 30% after 1 h of the addition of lincomycin (LINC), a chloroplast protein synthesis inhibitor, showing significant D1 degradation and repair under low irradiance. Exposure to HL lead to a 3.2-fold increase in D1 degradation rate, whereas average D1 repair rate was 1.3-x higher under HL than LL, leading to decreased levels of D1 protein under HL. There were significant effects of both HL and LINC on *P. tricornutum* maximum quantum yield of PSII (*F*_v_/*F*_m_), showing a reduction of active PSII reaction centres. Partial recovery of *F*_v_/*F*_m_ in the dark demonstrates the photosynthetic resilience of this diatom to changes in the light regime. *P. tricornutum* showed high allocation of total protein to D1 and an active D1-repair cycle to limit photoinhibition.

## Introduction

Diatoms (Heterokontophyta, Bacillariophyceae) are a major group of microalgae, ubiquitous in marine and freshwater ecosystems, contributing to approximately 20% of the global primary photosynthetic production [1]. Planktonic as well as benthic diatoms tend to dominate ecosystems characterized by unstable water bodies where they often have to cope with steep photic zone light gradients, fluctuating light regimes and punctuated exposures to high light (HL) that can be harmful for photosynthesis [2], [3].

In order to optimize growth and minimize photodamage, phototrophs have developed photoprotective mechanisms to cope with HL. One of the main physiological processes involved is the thermal dissipation of harmful excess energy through the xanthophyll cycle [4]. In diatoms, this cycle involves the de-epoxidation of the pigment diadinoxanthin (DD) to diatoxanthin (DT) under HL, triggered by acidification of the thylakoid lumen [5], [6]. DT causes ‘non-photochemical quenching’ (NPQ) in the antenna pigment-protein complexes, that decrease the excitation rate of PSII reaction centres. Ruban et al. [7] showed that *Phaeodactylum tricornutum* could form NPQ 3 to 5 times larger than higher plants, which may be a central feature explaining the success of diatoms in variable light environments [6]. Recently, Bailleul et al. [8] revealed that the levels of LHCX1, an atypical member of the light-harvesting complex stress-related protein family, are directly related to the ability of *P. tricornutum* to quench excess energy.

If photoprotective mechanisms are insufficient to counteract HL, overexcitation of PSII occur leading to the production of reactive oxygen species (ROS) and damage to the photosynthetic apparatus [9]. The reaction centre protein D1, that binds the primary donors and acceptors active in PSII electron transport, is the component of PSII most prone to photooxidative damage. Phototrophs have developed an unusually rapid D1 turnover repair cycle [10], involving proteolytic release of the damaged D1, *de novo* synthesis of the protein and the incorporation of new D1 into reassembled PSII complexes [11]. If photoinactivation exceeds the rate of repair of this protein, photoinhibition of photosynthesis occurs because the pool of active PSII reaction centres declines. Fast turnover of multiple PSII subunits and the induction of transcriptional processes involved in the protection of cellular structures have been shown in marine diatoms at an early phase of HL exposure [12], [13].

Photoprotection and photoinhibition of PSII in diatoms is typically investigated using rapid, non-invasive chlorophyll *a* fluorescence techniques [14], [15], [16], whereas direct analyses of D1 protein content using molecular detection tools are scarce [13]. Here, we quantitatively analyse the response of the pennate diatom *Phaeodactylum tricornutum* to HL exposure using a combination of complementary techniques: Pulse Amplitude Modulated (PAM) fluorometry, High Performance Liquid Chromatography (HPLC) pigment analysis and D1 protein immunodetection. D1 repair capacities were assessed using lincomycin (LINC), an inhibitor of chloroplast-encoded protein synthesis.

## Results

### D1 degradation and re-synthesis

**Figure 1:**
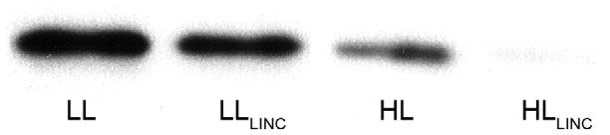
Immunodetection of the PSII reaction centre protein D1 in *Phaeodactylum tricornutum*. Treatments (1 h duration): LL (low light, 40 µmol photons m^−2^ s^−1^); HL (high light, 1,250 µmol photons m^−2^ s^−1^); LL_LINC_ (low light with LINC); and HL_LINC_ (high light with LINC).

**Figure 2:**
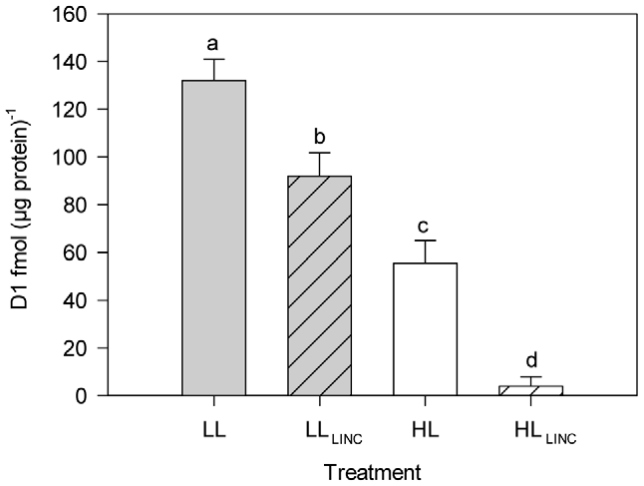
Protein D1 content in *Phaeodactylum tricornutum*. Treatments (1 h duration): LL (low light, 40 µmol photons m^−2^ s^−1^); HL (high light, 1,250 µmol photons m^−2^ s^−1^); LL_LINC_ (low light with LINC); and HL_LINC_ (high light with LINC). D1 *per* total protein (fmol µg^−1^; mean ± standard deviation, n = 7). Different letters indicate significant differences between treatments (*P*<0.001).

An example of D1 protein immunodetection is shown in Fig. 1. Exposure of *P. tricornutum* to HL resulted in a decrease in D1 content when compared to cells subjected to LL, particularly if chloroplast protein synthesis was inhibited by the addition of LINC (Figs. 1 and 2). D1 content in LL acclimated cells was 132±8.8 fmol µg^−1^ protein (Fig. 2). There were significant (*P*<0.001) effects of both irradiance and LINC in D1 content. Degradation of D1 occurred at LL in the presence of LINC (LL_LINC_), as seen by a significantly (*P*<0.001) lower content: 92±9.8 fmol µg^−1^ protein (Fig. 2). This corresponded to an average 30% decrease in D1 levels after 1 h of the addition of LINC and an estimated degradation rate ( = D1 (LL) – D1 (LL_LINC_)) of 40.1 fmol µg^−1^ protein h^−1^.

D1 degradation under HL was much more severe than in LL, as shown by the lower D1 levels at HL. When repair was inhibited in HL (HL_LINC_) only a residual amount of D1 was detected in the extracts (4±4 fmol µg^−1^ protein), representing a degradation of 97% of the LL pool (Fig. 2) and an estimated degradation rate ( = D1 (LL) – D1 (HL_LINC_) of 128.1 fmol µg^−1^ protein h^−1^. In the absence of LINC, D1 pools under HL also dropped significantly (*P*<0.001) to 56±9.5 fmol µg^−1^ protein, corresponding to 42% of the D1 protein under LL (Fig. 2). Considering the same degradation rates at HL and HL_LINC_ treatments, then re-synthesis rate under HL ( = D1 (HL) – D1 (HL_LINC_)) was 51.5 fmol µg^−1^ protein h^−1^. An average 3.2-fold increase in D1 degradation rates was observed in HL, whereas re-synthesis rate was in average 1.3-x higher comparatively to LL.

### Pigment analysis

**Figure 3:**
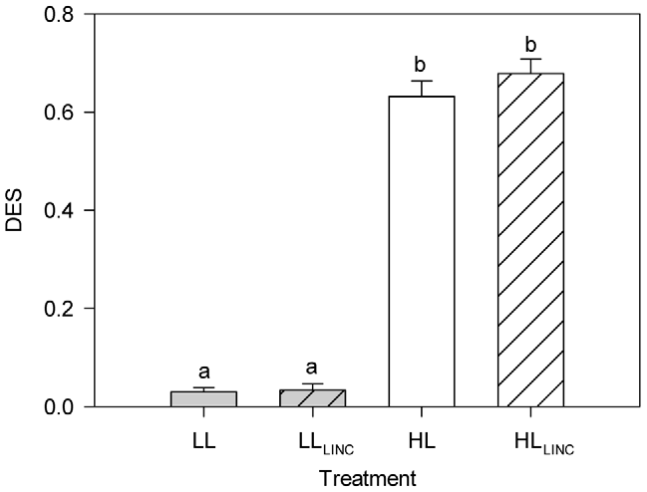
De-epoxidation state (DES) in *Phaeodactylum tricornutum*. Treatments (1 h duration): LL (low light, 40 µmol photons m^−2^ s^−1^); HL (high light, 1,250 µmol photons m^−2^ s^−1^); LL_LINC_ (low light with LINC); and HL_LINC_ (high light with LINC). Mean ± standard deviation (n = 4). Different letters indicate significant differences between treatments (*P*<0.001).

No significant effects of light treatment and LINC were observed in the concentrations of diatom major pigments such as fucoxanthin, chlorophyll *c_2_*, chlorophyll *a* (Chl *a*) and *β*-carotene (Table 1). A significant (*P*<0.001) effect of light treatment was observed in the concentrations of the xanthophyll cycle pigments diadinoxanthin (DD) and diatoxanthin (DT). Concentrations of DD were similar in LL and LL_LINC_ treatments but were significantly (*P*<0.001) lower under HL and HL_LINC_ (Table 1). DT concentrations followed an opposite trend, being extremely low under LL and being significantly (*P*<0.001) higher in both HL and HL_LINC_. DT concentrations showed a more than 20-fold change in concentration increasing from 1.3 fg cell^−1^ under LL to 30 fg cell^−1^ under HL (Table 1). Accordingly, the de-epoxidation state (Fig. 3) changed significantly (*P*<0.001) from LL (0.030±0.009 and 0.033±0.013 for LL and LL_LINC_ respectively) to HL (0.632±0.032 and 0.679±0.030 for HL and HL_LINC_ respectively). After 1 h of HL, approximately 62% of DD was converted to DT by the xanthophyll cycle. There was no significant effect of LINC in the concentrations of DD and DT. There was no significant (*P* = 0.094) effect of light treatment on the concentrations of DD+DT, although concentrations were slightly higher under HL than LL (Table 1).

**Table 1:** Pigment concentrations (fg cell^−1^) in *Phaeodactylum tricornutum*.

	LL	LL_LINC_	HL	HL_LINC_
Chl *a*	369±39^a^	347±25^a^	345±19^a^	340±41^a^
Fucoxanthin	206±12^a^	198±32^a^	199±14^a^	180±8^a^
Chl *c_2_*	65.6±8.3^a^	66.1±11.4^a^	67.5±5.5^a^	57.4±3.2^a^
*β*-carotene	16.0±1.1^a^	13.3±2.3^a^	13.8±5.3^a^	13.7±0.2^a^
DD	41.2±3.3^a^	41.3±2.6^a^	17.0±2.6^b^	14.7±0.4^b^
DT	1.3±0.4^a^	1.4±0.5^a^	29.0±3.1^b^	31.1±1.9^b^
DD+DT	42.5±3.6^a^	42.7±2.2^a^	46.0±5.4^a^	45.9±1.6^a^

Treatments (1 h duration): LL (low light, 40 µmol photons m^−2^ s^−1^); HL (high light, 1,250 µmol photons m^−2^ s^−1^); LL_LINC_ (low light with LINC); and HL_LINC_ (high light with LINC). Mean ± standard deviation (n = 4). Different letters indicate significant differences between treatments (*P*<0.001).

### Chlorophyll a Fluorescence

**Figure 4:**
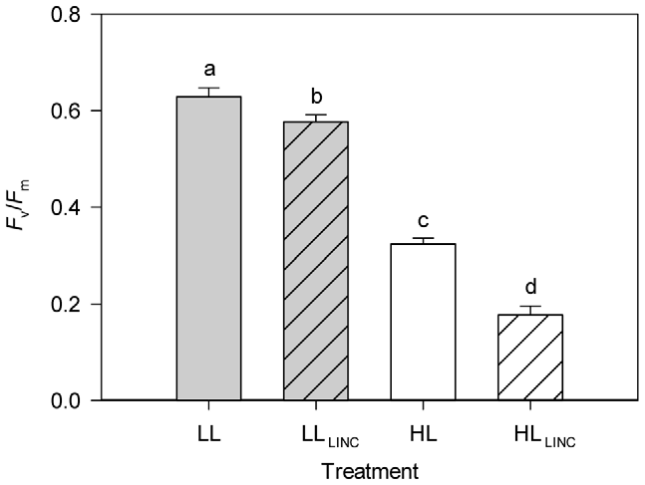
Maximum quantum yield of PSII (*F*_v_/*F*_m_) in *Phaeodactylum tricornutum*. Samples were dark-adapted for 10 min. Treatments (1 h duration): LL (low light, 40 µmol photons m^−2^ s^−1^); HL (high light, 1,250 µmol photons m^−2^ s^−1^); LL_LINC_ (low light with LINC); and HL_LINC_ (high light with LINC). Mean ± standard deviation (n = 4). Different letters indicate significant differences between treatments (*P*<0.01).

There were significant (*P*<0.001) effects of both irradiance and LINC in the maximum quantum yield of PSII (*F*_v_/*F*_m_) of 10 min dark-adapted samples (Fig. 4). For LL treatment, *F*_v_/*F*_m_ were significantly (*P*<0.01) lower in the presence of LINC (0.629±0.019 and 0.577±0.014 for LL and LL_LINC_ respectively). *F*_v_/*F*_m_ were significantly (*P*<0.001) lower in both HL treatments when compared to LL. For HL, *F*_v_/*F*_m_ were significantly (*P*<0.001) lower in the presence of LINC (0.323±0.013 and 0.177±0.019 for HL and HL_LINC_ respectively).

**Figure 5:**
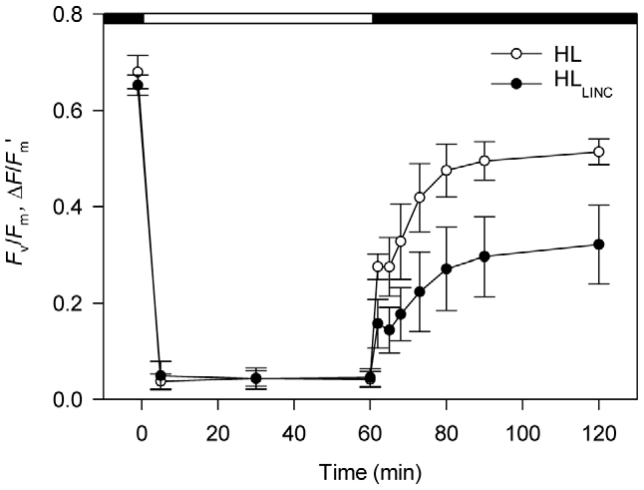
Variation in PSII maximum (*F*_v_/*F*_m_) and effective (Δ*F*/*F*_m_′) quantum yield in *Phaeodactylum tricornutum*. Measurements before and during exposure to 1 h of high irradiance (1,250 µmol photons m^−2^ s^−1^), and subsequent recovery in the dark. Treatments: HL (control samples); and HL_LINC_ (LINC inhibited samples). Mean ± standard deviation (n = 5).

Maximum (*F*_v_/*F*_m_) and effective (Δ*F*/*F*_m_′) quantum yield of PSII in *P. tricornutum* before and during HL and subsequent recovery in the dark are depicted in Fig. 5. Under HL, Δ*F*/*F*_m_′ was extremely low (∼0.05) and stable in both treatments. When transferred to the dark, after the HL period, values recovered gradually, but differently depending on the treatment. In the absence of inhibitor (HL), *F*_v_/*F*_m_ recovered after 1 h in the dark to 0.514±0.027. Compared with the initial *F*_v_/*F*_m_, it represented a 76% recovery. When the PSII repair cycle was blocked by the addition of LINC (HL_LINC_), recovery of *F*_v_/*F*_m_ was significantly lower, reaching 0.321±0.081 after 1 h in the dark. Recovery of *F*_v_/*F*_m_ was still visible between 30 min and 1 h in the dark in both treatments (HL and HL_LINC_).

**Figure 6:**
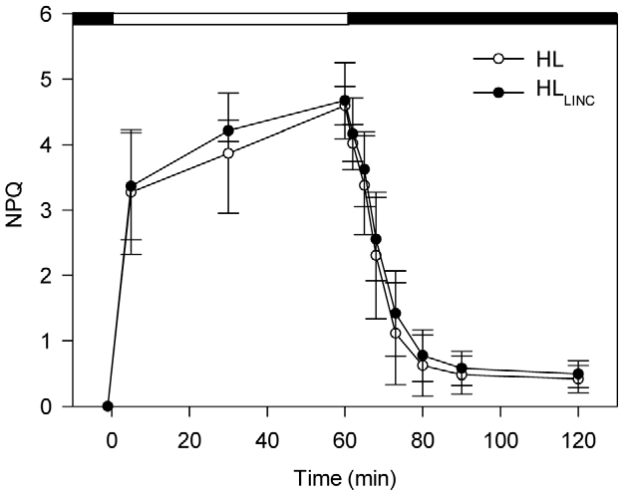
Variation of non-photochemical quenching (NPQ) in *Phaeodactylum tricornutum*. Measurements before and during exposure to 1 h of high irradiance (1,250 µmol photons m^−2^ s^−1^), and subsequent recovery in the dark. Treatments: HL (control samples); and HL_LINC_ (LINC inhibited samples). Mean ± standard deviation (n = 5).

Results on non-photochemical quenching in *P. tricornutum* during 1 h of HL and subsequent recovery in the dark are depicted in Fig. 6. When HL was applied, NPQ increased rapidly to values of approximately 3 after 5 min and gradually increased throughout the entire HL period in both treatments to *ca.* 4.6 after 1 h of HL. When transferred to the dark, after the HL period, NPQ decreased rapidly and similarly in control and LINC-treated cells. NPQ values reached slightly lower values under HL (0.42±0.21) than HL_LINC_ (0.49±0.20) after 1 h in the dark. Relaxation of NPQ was still visible between 30 min and 1 h in the dark in both treatments (HL and HL_LINC_), although less than the corresponding recovery of *F*_v_/*F*_m_.

## Discussion

The D1 protein is possibly the most researched photosynthetic polypeptide, tied up in a still controversial way with photoinhibition [17], [18]. In this study, significant effects of HL were observed in both D1 protein degradation and re-synthesis in *P. tricornutum*: degradation of D1 was 3.2-x higher under HL, while average re-synthesis was 1.3-x higher than in LL. This data is in accordance with Nymark et al. [12] who found specific up-regulation under HL of two *ftsH* genes encoding proteases functioning in the degradation of D1 protein and also two genes (*HCF136* and *PSB27*) encoding proteins involved in the assembly and repair of PSII in *P. tricornutum*. Furthermore, these authors observed that the transcription of *psbA* gene encoding the D1 protein was maintained in an early phase of HL exposure, when several genes encoding subunits of PSII were down-regulated. This indicates that short-term HL treatment caused increased photodamage in *P. tricornutum* and that mechanisms necessary for PSII recovery were in place [12].

D1 content *per* µg of total protein in *P. tricornutum* was higher than that reported for centric diatoms *Thalassiosira pseudonana* and *Coscinodiscus radiatus* (47±9 and 23±4 fmol µg^−1^ protein, respectively) [13] and for sea-ice diatoms (10–30 fmol µg^−1^ protein) [19]. However, D1 protein levels were similar to those of protein D2 found in *T. pseudonana* (121±3 fmol µg protein^−1^) [13], with which D1 forms the heterodimer core of the PSII reaction centre. Key et al. [20], studying a panel of seven centric marine diatoms of the genera *Thalassiosira* and *Coscinodiscus* with a cell volume span of 10^1^ to 10^7^ µm^3^, found that D1 *per* total protein increased with cell size. Considering that cells of *P. tricornutum* had a small average biovolume of 7.74×10^1^ µm^3^ [21], this diatom species allocates a particularly high proportion of its total protein to D1. This can constitute an advantage for this species when dealing with photoinhibitory light levels. Since most of the available data on D1 levels are for centric species, further work is needed to determine whether this is a particular characteristic of *P. tricornutum* or a more general feature of pennate diatoms.

The decrease in D1 content of control and LINC-treated cells of *P. tricornutum* under HL, found in this study, were comparable to the ones observed in the small diatom *T. pseudonana* [13] and in natural phytoplankton communities dominated by diatoms [22]. The former authors found no net loss of D1 in control cultures of the larger diatom *C. radiatus* and a less significant decrease in LINC-treated cells in comparison to our study. Although with significantly lower light levels applied, minimal effects of the addition of LINC on the photosynthetic performance of diatoms of the genera *Fragilariopsis* have been reported [16], [19], as well as on the temperate large marine centric diatom *Coscinodiscus wailesii* [20]. According to the latter authors, large diatom species show slower metabolic repair of PSII, but lower susceptibility to short-term exposure to HL due to their smaller effective cross-sections for photoinactivation in comparison to smaller diatoms.

Decrease in D1 protein levels in *P. tricornutum* was also observed in LL after the addition of LINC, showing significant D1 degradation (and re-synthesis) under low irradiance. Simultaneously, a decrease of *F*_v_/*F*_m_ was observed, showing a loss of active PSII reaction centres. In fact, Matoo et al. [10] reported that D1 protein degradation is a process largely associated with low photon fluences. Jansen et al. [23], following degradation of D1 in *Spirodela oligorrhiza* plants, found that light as low as 6 µmol m^−2^ s^−1^ elicited a reaction constituting more than 25% of the total degradation response.

Regarding the major light-harvesting pigments such as Chl *a* and fucoxanthin, short-term HL treatment had no significant effect on their cellular concentrations. However, concentrations of Chl *a* were slightly lower under HL (see Table 1), so we can speculate that longer exposure to HL would cause a reduction of *P. tricornutum* light harvesting pigments. Nymark et al. [12] observed a severe repression of nuclear-encoded genes involved in all steps of the Chl *a* metabolism in *P. tricornutum* within 30 min of exposure to HL, although not reflected in Chl *a* concentrations at this early stage of HL acclimation. A decrease in Chl *a* concentrations limits light harvesting and consequently energy transfer for photosynthesis, therefore protecting the photosynthetic apparatus from oxidative damage.

We observed a strong induction of NPQ and the activation of the xanthophyll cycle through the conversion of DD to DT in *P. tricornutum* under HL. In this study, NPQ values between 3 and 5 under high irradiances are similar to the ones previously reported for this species and considerably higher than those observed for higher plants [6], [7], [24]. The xanthophyll cycle is the most important short-term photoprotection mechanism in diatoms [6], [7], [12], reducing the light energy reaching PSII and the photoinactivation of the D1 protein. Lavaud et al. [24] found a significant increase in the pool of DD cycle pigments in *P. tricornutum* grown under intermittent light regimes, allowing cells to virtually eliminate photoinhibition by saturating light. This might explain the dominance of diatoms in variable light environments characteristic of turbulent waters [6]. Recently, Bailleul et al. [8] have shown that the peculiar characteristics of NPQ in diatoms are not due solely to the presence of the DD cycle and unveiled the key role of the protein LHCX1 as a molecular gauge controlling the levels of NPQ. The constitutive presence of this protein in *P. tricornutum* acclimated to nonstressful light conditions could provide cells with the capacity of anticipating sudden changes in light levels [8].

In our study, NPQ relaxation in *P. tricornutum* in the dark was fast and practically not affect by the addition of LINC and the consequent inhibition of PSII repair cycle. This indicates that NPQ, measured as the changes in (*F*_m_-*F*_m_′)/*F*_m_′, were mainly due to rapidly inducible and reversible quenching (qE), rather than photoinihibitory, slowly reversible quenching (qI) [9]. Furthermore, that increased photoinhibition in LINC-treated *P. tricornutum*, shown by lower D1 protein levels, increased *F*_0_ levels while practically not affecting *F*_m_. Regarding NPQ, our results relate to Lavaud et al. [25] who observed rapid relaxation of NPQ in the dark in *P. tricornutum* after exposure to HL. However, Grouneva et al. [26] showed that rapid DT epoxidation in this species occurs only under low light intensities and is severely restricted in the dark. The latter authors argue that this occurs due to shortage of NADPH, cosubstrate of DT epoxidase. The slower relaxation phase of NPQ in the dark and consequent recovery of *F*_v_/*F*_m_ observed in the presence of LINC, which cannot be attributed to D1 re-synthesis, can be explained by the NADPH-limited epoxidation of DT in complete darkness. After transition from HL to darkness the Calvin Cycle would act as a sink for NADPH, but the shortage of this cosubstrate would progressively decrease with deactivation of RuBisCO in the dark [27].

When cells of *P. tricornutum* were returned to the dark after short-term HL treatment, they showed partial recovery of PSII maximum quantum yield within 1 h. The greater recovery observed in control cells can be attributed to both relaxation of NPQ by the xanthophyll cycle and active PSII repair mechanisms. The limited recovery in samples treated with LINC is attributed exclusively to the conversion of DT back to DD as D1 repair was blocked. Despite sustained photoinhibition, the partial recovery of photochemical capacity after the HL period demonstrates the photosynthetic resilience of *P. tricornutum* to changes in the light regime.

The results of this study increase our understanding of the response mechanisms of diatoms to punctuated exposure to HL, underlying the importance of the xanthophyll cycle to increase the dissipation of excess light energy, but also of high allocation of total protein to D1 and active D1-repair to limit photoinhibition.

## Materials and Methods

### Algal cultures and experimental set-up

Monoalgal cultures of *Phaeodactylum tricornutum* Bohlin (IO 108-01, ALISU Algae Collection, Centre of Oceanography, University of Lisbon) isolated from samples from Ria de Aveiro (Aveiro, Portugal), were grown in f/2 medium [28] in a growth chamber (Fitoclima 250E, Aralab, Rio de Mouro, Portugal) at 15°C and 40 µmol photons m^−2^ s^−1^ irradiance (12∶12 h photoperiod). Cultures were grown in flasks and sampled at mid-exponential phase. Cells were counted by light microscopy (Olympus BX50, Tokyo, Japan) using a Neubaeur improved counting chamber, following staining with Lugol's iodine solution. A minimum of 400 cells was counted at a magnification of ×400. Cultures subjected to low light (LL) were maintained at the same growing conditions specified above, while high light (HL) stressed cultures were transferred to a Fytoscope FS130 (Photon Systems Instruments, Darsov, Czech Republic) for 1 h at 1,250 µmol photons m^−2^ s^−1^ and 15°C. Half of the samples in each light treatment were inoculated with LINC at a final concentration of 0.4 mg mL^−1^ (LL_LINC_ and HL_LINC_), an inhibitor of chloroplast-encoded protein synthesis, including D1. The inhibitor was added to the cultures 15 min before light stress.

### Protein extraction and immunodetection of D1

Total protein was extracted from 20 mL of algal culture, filtered using 25 mm GF/F Whatman filters and immediately frozen in liquid nitrogen. Microalgae were scratched off the filters to eppendorf tubes containing 1 mL of extraction buffer (sodium phosphate 10 mM, pH 7.4; EDTA 1 mM; 0.2% Tween 20 (v/v)) supplemented with freshly prepared phenylmethylsulfonyl fluoride (PMSF) 1 mM and dithiothreitol (DTT) 2 mM. Extracts were homogenized with a vortex and frozen in liquid nitrogen. Samples were then incubated at 80°C for 5 min, sonicated (Bransonic 220, Branson, Danbury, CT, USA) for 1 min and vortexed. The freeze-thaw cycle was repeated 4 times. To eliminate cell debris the samples were centrifuged at 10,000 *g* for 20 min at 4°C. Two additional extractions of the pellets detected only residual amounts of protein and the efficiency of the extraction method was 98.1±1.1%. Protein concentrations were determined with Bradford microassay (Bio-Rad, Hercules, CA, USA) using bovine serum albumin (BSA; Sigma-Aldrich, St. Louis, MO, USA) as a standard.

For each replicate, 2 µg of total proteins were separated by SDS-PAGE in a 12% polyacrylamide gel using the mini-protean 3 system from Bio-Rad. Different amounts of purified PsbA/D1 protein (Agrisera, Vännäs, Sweden) were also loaded in the gel in order to build a calibration curve to determine D1 concentrations in culture samples. A protein standard (Novex, Life Technologies, Carlsbad, CA, USA) was used to calculate protein size and control migration. Protein transfer to nitrocellulose membrane was performed in a Trans-Blot Semi-Dry Electrophoretic Transfer Cell (Bio-Rad) for 1 h at 140 mA in buffer (25 mM Tris, 192 mM glycine, 20% (v/v) methanol, pH 8.3). Protein loading was checked by Ponceau S staining. Membranes were blocked in PBS-T buffer supplemented with defatted powder milk (5% w/v) for 1 h at room temperature. A rabbit anti-PsbA antibody (Agrisera) was used for detection of D1, at 1∶20,000 dilution in blocking buffer for 1 h. Horseradish peroxidase (GE Healthcare, Little Chalfont, UK) coupled secondary anti rabbit IgG antibody was incubated for 1 h at a dilution of 1∶40,000. Four 15 min washings with PBS-T were performed after antibody incubation. Chemoluminescence detection was done using ECL Advance Western Blotting Detection Kit (GE Healthcare). Developed films (Hyperfilm ECL, GE Healthcare) were imaged with a Gel Doc XR imaging system to quantify band intensities by densitometry, using Quantity-One software (all Bio-Rad).

### Pigment analysis by HPLC

Pigments were extracted from 10 mL of algal culture, rapidly filtered using 25 mm GF/F Whatman filters and immediately frozen in liquid nitrogen. Filters were homogenizing in 95% cold buffered methanol (2% ammonium acetate) using a glass rod. Samples were then sonicated for 30 s, briefly vortexed and transferred to −20°C for 30 min. Supernatants were collected after centrifugation at 1,100 *g* for 5 min at 4°C, and filtered through 0.2 µm Fluoropore membrane filters (Millipore, Billerica, MA, USA). Extracts were then immediately injected into a HPLC system (Shimadzu, Kyoto, Japan) with a photodiode array detector (SPD-M10AVP). Chromatographic separation was carried out using a Supelcosil C18 column (25 cm long; 4.6 mm in diameter; 5 µm particles; Sigma-Aldrich, St. Louis, MO, USA) for reverse phase chromatography and a 35 min elution programme. The solvent gradient followed Kraay et al. [29], with an injection volume of 100 µL and a flow rate of 0.6 mL min^−1^. Identification and calibration of the HPLC peaks was done using pigment standards from DHI (Hørsolm, Denmark). Pigments were identified from absorbance spectra and retention times and their concentrations were obtained from the signals in the photodiode array detector. The de-epoxidation state (DES) was calculated as DES = DT/(DD+DT), where DT and DD are the concentrations of diatoxanthin and diadinoxanthin, respectively.

### PAM fluorometry

From each replicate, 1.2 mL of algal culture were transferred to a liquid-phase Clark-type oxygen electrode chamber (DW2/2 electrode chamber, Hansatech Instruments Ltd., Norfolk, UK) and kept homogeneous by constant mixing. Temperature was maintained at 15°C by a circulating water bath (Haake C10-K10, Thermo Fisher Scientific, Waltham, MA, USA). Fluorescence measurements were performed using a PAM 101 fluorometer (Walz GmbH, Effeltrich, Germany) connected to a PAM Data Acquisition System PDA 100 (Walz GmbH, Effeltrich, Germany). An external light source (KL 1500 LCD, Schott AG, Mainz, Germany) was used to provide the saturating light pulses. Maximum quantum yield of PSII (*F*_v_/*F*_m_ = (*F*_m_-*F*_0_)/*F*_m_) was determined after 10 min of dark incubation, where *F*_m_ and *F*_0_ are the maximum and minimum fluorescence of dark-adapted cells, respectively.

HL treatment (1,250 µmol photons m^−2^ s^−1^) was also simulated directly in the electrode chamber using an external light source to provide the actinic light (KL 1500 LCD, Schott AG, Mainz, Germany). *F*_v_/*F*_m_ was measured in dark-adapted intact cells before the onset of HL and after the light stress period (at 2, 5, 8, 13, 20, 30 and 60 min). PSII effective quantum (Δ*F*/*F*_m_′ = (*F*_m_′-*F*_s_)/*F*_m_′) was measured during HL (at 5, 30 and 60 min), where *F*_m_′ and *F*_s_ are maximum and steady-state fluorescence of light-adapted cells, respectively. Non-photochemical quenching (NPQ) was calculated as NPQ = (*F*_m_-*F*_m_′)/*F*_m_′, where *F*_m_ is the maximum fluorescence measured in dark-adapted intact cells before the onset of HL and *F*_m_′ is the maximum fluorescence of cells illuminated by HL or exposed to periods of darkness following HL.

### Statistical Analysis

The existence of significant differences in D1 protein levels, pigment concentrations and *F*_v_/*F*_m_ was tested using two-way analysis of variance (ANOVA) for effects of light and protein synthesis inhibitor (LINC). Data normality and homogeneity of variances were tested with Shapiro-Wilk and Bartlett tests, respectively. Data was transformed whenever necessary to comply with ANOVA assumptions. Post-hoc comparisons were made with Tukey HSD tests. All statistical analyses were carried out using Statistica 10.0 (StatSoft Inc., Tulsa, OK, USA).
